# Study on MRI Changes in Phenylketonuria in Patients Referred to Mofid Hospital/Iran

**Published:** 2014

**Authors:** Parveneh KARIMZADEH, Farzad AHMADABADI, Narjes JAFARI, Fakhreddin SHARIATMADARI, Hamid NEMATI, Adel AHADI, Sanaz KARIMI DARDASHTI, Mehrdad MIRZARAHIMI, Zahra DASTBORHAN, Javad ZARE NOGHABI

**Affiliations:** 1 Pediatric Neurology Research Center, Shahid Beheshti University of Medical Sciences, Tehran, Iran; 2 Pediatric Neurology Department, Mofid Children Hospiutal, Faculty of Medicine, Shahid Beheshti University of Medical Sciences, Tehran, Iran; 3 Pediatric Neurology Department, Ardabil University of Medical Sciences, Ardabil, Iran; 4 Pediatric Neurology Department, Arak University of Medical Sciences, Arak, Iran; 5 Pediatric Department, Ardabil University of Medical Sciences, Ardabil, Iran; 6 Surgery Department, Tehran University of Medical Sciences, Tehran, Iran; 7 Department of Neonatology, Ardabil University of Medical Sciences, Ardabil, Iran; 8 Ophthalmology Department, Isfahan University of Medical sciences, Isfahan, Iran; 9 Pediatric Nephrology Department, Ardabil University of Medical Scinces, Ardabil, Iran

**Keywords:** Phenylketonuria, MRI, Thompson score

## Abstract

**Objective:**

Phenylketonuria is one of the most common metabolic disorders and the first known cause of mental retardation in pediatrics. As Screening for phenylketonuria (PKU) is not a routine neurometabolic screening test for neonates in Iran, many PKU cases may be diagnosed after developing the clinical symptoms. One of the findings of PKU is myelination disorders, which is seen as hypersignal regions in T2-weighted (T2W) and FLAIR sequences of brain MRI. The aim of our study was to assess MRI changes in PKU patients referred to Mofid Children’s Hospital, 2010-2011.

**Materials & Methods:**

We studied all PKU cases referred to our clinic as a referral neurometabolic center in Iran for brain MRI and assessed the phenylalanine level at the time of Imaging. The mean phenylalanine level (in one year), clinical manifestations, and MRI pattern based on Thompson scoring, were evaluated.

**Results:**

The mean age of our study group was 155±99 months and the mean diagnosis age was 37±27.85 months. There were 15 patients with positive and 15 with negative family history. The mean phenylalanine level at the time of imaging was 9.75±6.28 and the mean 1 year phenylalanine level was 10.28±4.82. Seventy percent of our patients had MRI involvement, in whom 20% showed atrophic changes, in addition to white matter involvement. Based on modified Thompson scoring, the score for our study group was 4.84.

The maximum involvement in MRI was in occipital region, followed by parietal, frontal, and temporal zones. There was not any correlation between MRI score and patients’ age. But we found significant relationship between MRI score and the age of regimen cessation. No correlation was seen between phenylalanine level (at the time of Imaging) and MRI score. But there was a relationship between mean 1 year phenylalanine level and MRI score.

**Conclusion:**

According to the results of this study, brain MRI and white matter involvement can be used for evaluation of long-term control of phenylalanine level in PKU cases.

## Introduction

Phenylketonuria (PKU) is the first known cause of mental retardation, the first known metabolic disorder, and the first treatable inborn error of metabolism (1, 2).

Although, we have prenatal diagnosis of PKU and also diagnosis by screening methods in the first days of life, many of cases remains undiagnosed until they become symptomatic.

Because the screening methods for measurement of phenylalanine level were not routine in our country, many of cases are diagnosed after developing clinical symptoms.

PKU is characterized by mental retardation, microcephaly, blond hairs, and specific odor caused by the presence of keto acids in urine (3-6). 

Severity of MRI findings (white matter involvement) correlates with mean phenylalanine level in the past years and point phenylalanine level at the time of imaging (7, 8).

Many studies showed correlation between phenylalanine level and MRI white matter involvement in conventional methods and with a higher sensitivity in DWI (9, 10). 

In the present study, we aimed to evaluate the correlation between white matter involvement in T2-weighted (T2W) and FLAIR sequences of brain MRI in our patients and its relationship with clinical symptoms and demographic parameters. 

## Materials & Methods

In this cross-sectional study, all PKU cases referred to Neurology clinic of Mofid Children Hospital during the December 2010-September 2012 were enrolled. A questionnaire containing personal information of patients, time of diagnosis of disease, time of diet initiation, developmental status, mean phenylalanine level in the past year, the point phenylalanine level of patient at the time of imaging, and clinical manifestations of the disease was filled in. Phenylalanine level was measured by high-performance liquid chromatography (HPLC) method in a reliable laboratory center.

Brain MRI (T1W-T2W-FLAIR) was obtained for each patient. All of imagings were performed using a 1.5 Tesla scanner (Siemens) by conventional protocols in coronal, axial and sagittal view (9-11).

White matter involvement was scored based on modified Thompson scoring (Table1) by an expert neuroradiologist (12).

All collected data were analyzed by t-test and Spearman’s and Pearson’s Correlation coefficients using SPSS 18. 

**Table 1:** Thompson scoring based on anatomic involvement

**Grade**	**Scan Appearence**	**Grade**	**Scan Appearence**
0	Normal	3	30-50% White matter involved
1	<10% White matter involved	4	50-75% White matter involved
2	10-30% White matter involved	5	>75% White matter involved

Six different part (frontal, parietal, temporal, occipital, brain stem, and other parts) of each hemisphere of brain were evaluated for white matter involvement and the involvement was graded 1

## Results

We studied 30 patients with PKU who were referred to neurology clinic of Mofid Children’s Hospital. The mean age of our study group was 155±99 months, with minimum age of 10 and maximum age of 312 months. 

Thirteen patients (43%) were female and 17 (57%) were male.

The mean age of diagnosis in our study was 37±27.85 months, with minimum age of 1 month and maximum age of 168 months. 

The mean age of diet initiation was 44.61±33.95 months. 15 Patients (50%) had positive family history of PKU and the rest had negative.

Consanguineous marriage was seen in parents of 20 cases (66.7%).

In our study group, the mean age for neck holding was 5.4±4.26 months, for sitting 11.4±7.76 months and for walking 19.43±6.13 months.

Mean phenylalanine level at the time of imaging (MRI) was 9.75±6.28 (range, 0.5- 23) mmol/dL. 

The mean phenylalanine level in the past 1 year was 10.28±4.82 mmol/dL. 

Twenty-one patients (70%) had MRI involvement and 9 patients had no pathologic changes in their MRI.

Atrophic cortical changes were seen in 6 (20%) cases of our study group. 

Based on FLAIR and T2W sequences in MRI of our study group, the mean Thompson score was 4.84±4.63 (range, 0-18).

The maximum white matter involvement was seen in occipital region (63.3%), followed by parietal lobe (43.3%). The least involvement was seen in temporal area (3.3%).

Ten patients of our study group (33.3%) had seizure, which was generalized tonic clonic in 60% of them, and complex partial seizure in 2 cases. Infantile spasm and myoclonic seizure each one was seen in 1 case. 

Eleven (36.7%) patients had abnormal EEG. About psychological symptoms, autistic features were seen in 7 cases (23.3%) and ADHD in 4 patients.

## Discussion

The mean age of diagnosis in our study was 37±27.85 months and this was a failure for our case finding system. The diet therapy must be started within the first week of life to prevent neurological and cognitive disturbances. This shows the importance of screening tests for the disease.

Unfortunately, in some cases, in spite of positive family history, PKU was diagnosed with obvious delay.

In most previous studies, the age of diagnosis was more than 10 years.

20 cases (62.7%) of our study group were products of consanguineous marriage, which shows the importance of screening tests in this group. 

The mean age for starting low phenylalanine diet was 44.61±33.95 months, which shows a significant delay between diagnosis and diet initiation in our patients. 

Evaluation of motor and language development showed that all developmental millstones happened with delay in our study group. Speech delay was more sever. 

The mean phenylalanine level over the past year in our patients was lower than former studies (3-5). As this study was done in pediatric patients, most of our cases were under low phenylalanine diet and their phenylalanine level were lower than previous studies with wider age distribution (4-6).

White matter involvement was seen in 21 (70%) cases and this was less than other studies (4,7). This may be due to higher phenylalanine level in previous studies. 

On the other hand, some of prior studies had been designated based on DWI, which is more sensitive than conventional MRI imaging causing better detection of white matter involvement (5).

In our study, the maximum white matter involvement was in occipital region and spreads to parietal and frontal areas in higher phenylalanine levels. This is compatible with previous studies (11,12).

MRI score (Thompson) had no correlation with patients’ age (p=0.176) similar to previous studies (4-6). MRI score had no significant relation with the age of diagnosis (p=0.369), which was consistent with other studies (7,8).

Our study shows more time from regimen cessation and more sever and extensive MRI involvement. Our study proved that there is a linear relationship between MRI score and age of regimen cessation (p=0.006).

We could not find any correlation between MRI score and present phenylalanine level (p=0.265). This is contrary to findings of previous studies, showed statistical correlation between MRI score and point phenylalanine level at the time of imaging (9-12).

This may be due to uctuation in phenylalanine level in our studied group, which caused difference between point phenylalanine level at the time of imaging and mean 1 year phenylalanine level. 

There is statistical correlation between MRI involvement and mean 1 years phenylalanine level of patients (p=0.017), which is compatible with previous studies.

**In conclusion, **considering cultural and economical limitation and trammels, assessment of mean 1 year phenylalanine level is the best indicator of white matter involvement and has more relation with MRI score compared to point phenylalanine level at the time of imaging. Therefore, we can use brain MRI and white matter involvement for evaluation of long-term control of phenylalanine level in PKU cases.
